# Surface-Enhanced Raman Scattering Active Core-Shell Ag NPs@Carbon Dots with Enzyme-Mimicking Activities for Label-Free Measurement Cholesterol

**DOI:** 10.3390/bios13100927

**Published:** 2023-10-16

**Authors:** Jian Ju, Lin Li, Bei Li, Sagar Regmi, Tingting Wang, Jiao Xu, Chaojie Li, Shixing Tang

**Affiliations:** 1 Wenzhou Institute, University of Chinese Academy of Sciences, Wenzhou 325001, China; li18367819219@163.com (L.L.); wangtingting@ucas.ac.cn (T.W.); xujiao@ucas.ac.cn (J.X.); lichaojie@ucas.ac.cn (C.L.); 2 Oujiang Lab, Wenzhou 325001, China; 3 School of Ophthalmology & Optometry, Wenzhou Medical University, Wenzhou 325035, China; beili@ciomp.ac.cn; 4 The State Key Lab of Applied Optics, Changchun Institute of Optics, Fine Mechanics and Physics, Chinese Academy of Sciences, Changchun 130033, China; 5 Department of Pharmacology, School of Medicine, Case Western Reserve University, Cleveland, OH 44106, USA; sagar005@e.ntu.edu.sg; 6 Department of Epidemiology, School of Public Health, Southern Medical University, Guangzhou 510515, China

**Keywords:** cholesterol, doped carbon dots, silver nanocomposite, peroxidase-mimicking, Surface-enhanced Raman Spectroscopy (SERS), label-free assay

## Abstract

Serological-sensitive testing of cholesterol holds significant value in the fields of healthcare and clinical diagnosis. This study reports on the preparation of peroxidase-mimicking nanozymes through the wrapping of N, S-doped carbon dots (DCDs) on the surface of silver nanoparticles (Ag NPs@DCD). The shell–core structure of Ag NPs@DCD displays peroxidase-mimicking capability, with the potential to catalyze inactive Raman probe molecules into the Raman reporters. Furthermore, a “shell-isolated nanoparticles-enhanced Raman spectroscopy” structure exhibited an enhanced Raman signal of reporter molecules. Ag NPs@DCD were utilized to create a label-free SERS sensing system for high-performance detection of cholesterol in serum samples. These results demonstrate the potential of the novel nanozyme-based SERS approach for clinical diagnosis.

## 1. Introduction

Cholesterol (C_27_H_46_O) is a waxy substance that serves multiple functions such as building healthy cells as well as regulating cell membrane permeability and fluidity biomarkers of ischemic heart disease (IHD) and ischemic stroke (IS) [1,2]. In addition to that, some epidemiologic studies suggested a correlation between the risks of several malignancies and serum cholesterol levels [3,4]. It is generally accepted that the optimum concentration of cholesterol is in the range of 3 to 6 mM in human serum [5]. The abnormal cholesterol level often serves as a warning for several diseases such as hyperthyroidism, malnutrition anemia and low lipoprotein, etc. [6,7,8]. Therefore, the accurate monitoring of blood cholesterol in the body is important for health-related problems as well as clinical diagnosis.

At present, there are many methods to monitor the quantification of cholesterol, such as electrochemical [9,10,11], piezoelectric [12], colorimetric [13,14], fluorometric [11], surface plasmon resonance [15] and enzymes methods [10,16,17]. However, these technologies have some shortcomings, such as poor stability of electrochemical electrodes [18], susceptibility of colorimetric methods to color interference [19], photobleaching and easy quenching of fluorescent light-emitting groups [20]. Recently, a cholesterol biosensor based on Surface-enhanced Raman Spectroscopy (SERS) has been developed due to its rapid analysis, low small sample requirement, non-destructive properties, and high sensitivity. Wu et al. fabricated a shell–core nanoparticles with an embedded standard used as the novel SERS substant for detection of cholesterol in solution as well as inside the live cell at a single-cellular level [21]. He et al. prepared a kind of Ag NPs@MOF-based SERS-active sensor for high-performance detection of cholesterol in blood samples [22]. In this study, the Ag nanoparticles were grown on the surface of MIL-101 (Fe) to create the hybrid nanomaterial of AgNPs@MOF. This material is capable of oxidizing leucomalachite green (LMG) into the Raman-active malachite green (MG) in the presence of H_2_O_2_ from cholesterol oxidase (ChOx) during the oxidation of cholesterol. Interestingly, such a novel hybrid SERS biosensor can achieve high sensitivity and provide a more user-friendly strategy by reducing the operation steps to immobilize enzymes on the substrate surface. These articles propose novel strategies for developing efficient composite materials possessing both enzymatic activity and SERS-active properties, which could promote the use of SERS biosensors in vitro diagnostics application.

The sliver nanoparticles (Ag NPs) possess extraordinary SERS activities, which present great localized electromagnetic (EM) field enhancement [23]. The EM phenomenon is linked to the “hot spots” created via Localized Surface Plasmon Resonance (LSPR) on the surface of metals, such as gold (Au) or silver (Ag). The Raman signal experiences significant amplification when once the analyte molecules come close to the “hot spot” field [24]. Currently, researchers have reported abound of materials loaded with Ag NPs to obtain homogeneous structures, such as metallic oxide [25], MOF [22], carbon materials [26,27], polymer [28,29] and silica [30,31], which are conductive to improving the stability of Ag NPs and generating a large number of “hot spots”. Among the above-mentioned SERS-active substrates, the carbon-based materials are of particular interest as the Raman signals of target molecules can be enhanced through both chemical enhancement (CE) and EM by combining with plasmonic nanoparticles [32]. Zheng et al. [33] demonstrated that Graphene/Ag nanomaterials can generate numerous “hot spots” for the development of effective SERS biosensors. Meanwhile, Shi et al. reported on a two-steps approach to synthesize Graphene/Ag@ molecularly imprinted polymer (MIP) hybrid nanomaterial with outstanding SERS activity [32]. Furthermore, Ag NPs are receiving considerable interest as nanozymes due to their excellent stability, customizable catalytic performance, affordability and ease of preparation. With these advantages, silver nanozymes are being increasingly used in biosensing [34,35] and biomedicine [36]. However, creating ideal nanozymes that exhibit not only good catalytic activity but also excellent sensitivity for use in SERS sensor remains a challenge.

**Scheme 1:**
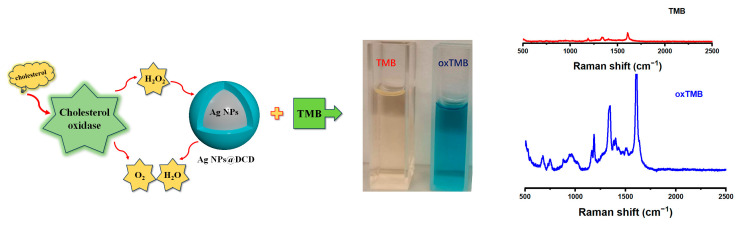
Illustration of the Ag NPs@DCD based on SERS biosensor to detect cholesterol.

In our study, we proposed a TMB-AgNPs@carbon dot-based SERS sensor probe for the first time. The substrate was prepared through a green and low-temperature technique using N, S-doped carbon dot (DCD) and Ag nanoparticles. The resulting structure features Ag NPs as the core and DCD as the shell, enabling a “shell-isolated nanoparticle-enhanced Raman spectroscopy” (SHINERS) effect. SHINERS technology was first reported by Prof. Tian’s group [37]. The method utilized shell-isolated nanoparticles to prevent direct contact between the core of the nanoparticle and the target molecules, resulting in enhanced Raman signals. Currently, SHINERS technology is being applied in various fields such as single crystal interfaces, food safety, cultural heritage, biological imaging, and photothermal therapy [38]. In addition, this substrate has a higher capacity to enrich molecules in its “hot spot”. In particular, Ag NPs@DCD exhibited a peroxidase-like activity through the increased oxidation of chromogenic 3, 3′, 5,5′-Tetramethylbenzidine (TMB) with H_2_O_2_. Utilizing oxTMB as Raman signal molecules, the TMB-Ag NPs@DCD probe effectively detected serum cholesterol concentration with speed and precision, as shown in Scheme 1.

## 2. Materials and Methods

### 2.1. Chemical Reagents

Citric acid (CA), thiourea (TU), 3, 3′, 5,5′-tetramethylbenzidine (TMB), ethanol, hydrogen peroxide (H_2_O_2_), Triton X-100 and sodium hydroxide (NaOH), Hydroxylamine hydrochloride (HH), silver nitrate, cholesterol oxidase (ChOx), uric acid (UA), cholesterol esterase (ChE), ascorbic acid (AA), urea, MgCl_2_ and dopamine (DA) were purchased from Aladdin Shanghai, China. NaAc-HAc buffer (0.2 M, pH 4.26), and PBS buffer (0.1 M, pH 7.0) were also used. Sigma Aldrich (Saint Louis, MO, USA) provided Rhodamine 6G (R6G) and Urea. Rabbit serum was provided by the Animal Experiment Centre of Wenzhou Institute, University of Chinese Academy of Sciences, China.

### 2.2. Instruments

The morphology and size images of N, S-doped carbon dot (DCD), Ag NPs and Ag NPs@DCD were analyzed using a high-resolution transmission electron microscope (HRTEM, FEI Talos F200S, Hillsbor, OR, USA). Raman spectra were measured using a Raman spectrometer with a 785 nm laser excitation wavelength (Renishaw, London, UK). A UV-vis spectrometer (TU-1901, Beijing, China) was employed to perform the UV-Vis absorption spectrum readings. Absorbance was quantified using an Epoch Microplate Spectrophotometer (Bio-Tek, Winooski, VT, USA, country).

### 2.3. Preparation of N, S-Doped Carbon Dot (DCD)

DCDs were synthesized via the hydrothermal method using a TU and CA mass ratio of 2:1. The resulting powder was dissolved in DMF solution and heated to 160 °C in an autoclave for 6 h [39]. After cooling to the room temperature, the products were collected via the centrifugation process (10,000 rpm for 10 min). Next, 5 mL of the obtained supernatant obtained was added dropwise to 40 mL of NaOH solution (50 mg/mL), and DCD (2.0 mg/mL) was then stored in a refrigerator at 4 °C.

### 2.4. Details the Preparation of Ag NPs and Ag NPs@DCD

Ag NPs were prepared using the method reported by Liu’s lab [40]. First, 1 mL of 10 mg mL^−1^AgNO_3_ was dropped into water (44 mL), and then 5 mL solution containing Triton X-100 (35 μL), HH (15 mM) and NaOH (30 mM) was added immediately. The mixture was then sonicated for 30 min at room temperature, and the resulting Ag NPs were purified via centrifugation and washed twice for 10 min at 10,000 rpm.

Following our previous work [41], Ag NPs@DCD were synthesized with a similar approach that was applied to pure Ag NPs. Briefly, 4 mL of DCD (10 ug/mL) was added and mixed before the introduction of Triton X-100 (35 μL), HH (15 mM) and NaOH (30 mM) and subjecting it to an ultrasonic treatment for 30 min. Finally, we washed the Ag NPs@DCD with centrifugation (10,000 rpm, 10 min) to remove unused DCD. The concentration of the reserve solution was adjusted to 2 mg mL^−1^.

### 2.5. Evaluation of Enzyme-Activity of AgNPs@DCD Nanocomposites

The mimetic peroxidase activity of Ag NPs@DCD was assessed by utilizing TMB and H_2_O_2_ as substrates. In the reactive system, various concentration of H_2_O_2_ and TMB were mixed to a HAc-NaAc buffer (0.2 M, pH 4.26) containing 0.25 mg mL^−1^ of Ag NPs@DCD solution. After incubation for 10 min, the absorbance of oxTMB at 652 nm was recorded on an Epoch Microplate Spectrophotometer.

### 2.6. Examination of SERS Activity of Prepared Ag NPs@DCD as SERS Substrate

The SERS activity of pure Ag NPs and Ag NPs@DCD was investigated using R6G as a Raman reporter. Specifically, 50 μL each of Ag NPs or Ag NPs@DCD (2 mg/mL) was added into R6G (100 μL, 10^−6^ M) and incubated for 5 min. Then, the SERS measurement was performed at a 785 excitation wavelength with an integration time of 10 s.

### 2.7. SERS Detection of Cholesterol Utilizing TMB-Ag NPs@DCD System

In this experiment, 0.1 M of cholesterol stock solution was prepared using an ethanol solution in a water bath at 60 °C. The above solution was then diluted to series concentration (2 to 200 μM) with a PBS solution. For a typical assay, a mixture of 10 μL of cholesterol sample and 50 μL of ChOx (70 UN mL^−1^) were incubated in PBS buffer (140 μL) at 37 °C for 30 min. Then, an aqueous solution of Ag NPs@DCD (0.25 mg mL^−1^) was mixed with 2 mM of TMB, and the resulting mixture was diluted with HAc-NaAc buffer (0.2 M, pH 4.26) to a final volume of 1 mL. The system was incubated for additional 30 min at 37 °C, and the Raman intensity at 1605 cm^−1^ was measured to detect cholesterol. The procedure of the selectivity experiment to detect cholesterol was the same as described above. The excitation wavelength for the Raman measurement was 785 nm with a power density of 1.2 W/mm^2^ and an exposure time of 10 s.

### 2.8. Testing of Serum Samples

To evaluate the application of the novel TMB-Ag NPs@DCD SERS biosensor, rabbit blood was chosen as a complex sample for detecting total cholesterol. To produce the free cholesterol, 50-fold serum samples (with PBS buffer) were mixed with 100 μL of 10 U mL^−1^ cholesterol esterase and 50 μL of 70 UN mL^−1^ cholesterol oxidase and incubated for 30 min at 37 °C [42]. Each sample was then mixed with the same concentration as mentioned before for Ag NPs@DCD, H_2_O_2_ and TMB. The mixture was incubated at 37 °C for 30 min and then employed for the SERS detection.

## 3. Result and Discussion

### 3.1. Characterization of DCD, Ag NPs and Ag NPs@DCD

**Figure 1:**
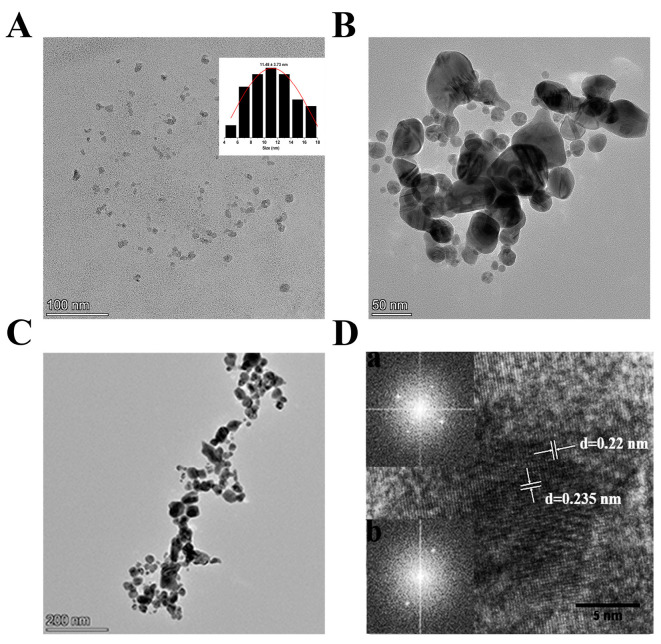
TEM images of DCD (**A**), Ag NPs (**B**) and Ag NP@DCD (**C**). Inset of the (**A**,**C**) show the size histograms of the DCD and Ag NPs@DCD, respectively. (**D**) HRTEM images of Ag NP@DCD and FFT patterns of DCD and Ag NPs@DCD shown in inset a and b respectively.

**Figure 2:**
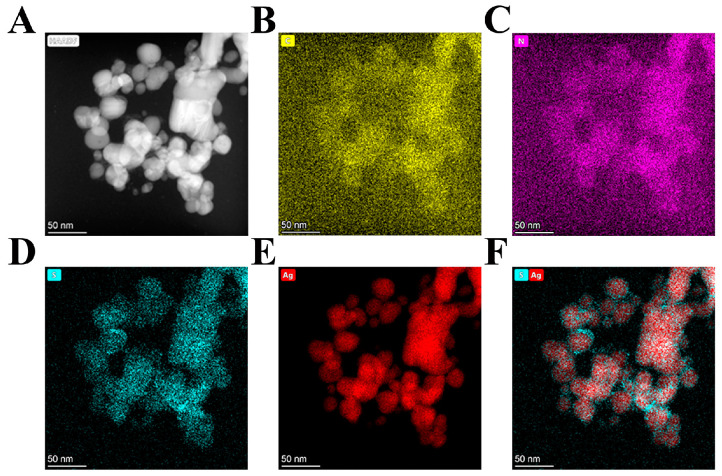
(**A**) EDX maps image of Ag NPs@DC, (**B**) C, (**C**) N, (**D**) S, (**E**) Ag and (**F**) overlapped image of Ag and S element.

The morphologies of the DCD, Ag NPs and Ag NPs@DCD were analyzed using TEM, as illustrated in Figure 1. DCD has a narrow size distribution of approximately 11 nm, as shown in Figure 1A. TEM image of Ag NPs with a relatively broad size distribution, and an apparent process of gradual fusion of small particle size silver nanoparticles into large particle size was observed in Figure 1B due to the weak structure control agent of TX-100 surfactant. However, in the presence of DCD, Figure 1C,D show that the capped Ag NPs obtained with DCD have a well-defined morphology with an average diameter of about 87 nm, as judged from the image analysis of 100 individual particles. These TEM images prove that DCD is coated on the surface of silver nanoparticles and has a significant control effect on morphology and structure of Ag NPs. High crystallinity, the fast Fourier transform (FFT) shows lattice spacing of 0.22 nm and 0.235 nm, which corresponds to the (1120) lattice fringes of graphitic carbon, and the (1 1 1) planes of Ag NP, respectively. This result indicated that DCD was successfully wrapped on the Ag NPs surface. The nanocrystals of the AgNPs@DCD were also analyzed via Scanning TEM (STEM) coupled with energy dispersive X-ray spectroscopy (EDX). Figure 2 displays an overlay of DCD and silver EDX maps, which demonstrates a distinct separation of the C, N, and S elements from carbon dots that are homogeneously distributed on the silver element, thus confirming the shell–core structure.

**Figure 3:**
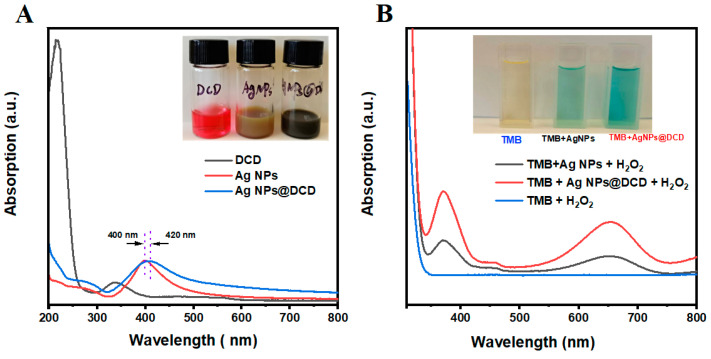
(**A**) UV–vis absorption spectra of DCD, Ag NPs and Ag NPs@DCD. Inset (**A**) shows the samples image as prepared in the bottle (from left to right: DCD, Ag NPs and Ag NPs@DCD). (**B**) UV–vis absorption spectra of TMB (curve blue line), oxTMB containing Ag NPs (curve black) and Ag NPs@DCD (curve red) in the presence of H_2_O_2_, respectively. The inset (**B**) shows digital photographs of the corresponding color changes.

The absorption spectra (UV–visible) of the aqueous dispersion of the DCD, Ag NPs, and Ag NPs@DCD are displayed in Figure 3A. A characteristic absorption peak attributed to the π-π* transitions in the core and the surface states is evident at 230 nm and 332 nm [32]. The pure Ag NPs synthesized via ultrasonication exhibit a typical peak at 400 nm (curve red), and then as expected, the peak of Ag NPs capped with DCD (curve blue) is observed at 420 nm, indicating an approximately 20 nm redshift. It is noted that encapsulating the Ag NPs with DCD causes the surface plasmon resonance (SPR) band to gradually shift towards longer wavelengths and become broader. This can be attributed to the formation of nanoparticle composites with more uniform and improved particle sizes, as well as the efficient plasmon-induced interfacial charge transfer (CT) processes (Ag to DCD CT transition) in AgNPs@DCD [43]. Based on the HRTEM and UV results, it can be concluded that Ag NP@DCD comprising a core–shell structure has been successfully prepared [35].

### 3.2. Examination of Raman Performance of Ag NPs and Ag NPs@DCD

**Figure 4:**
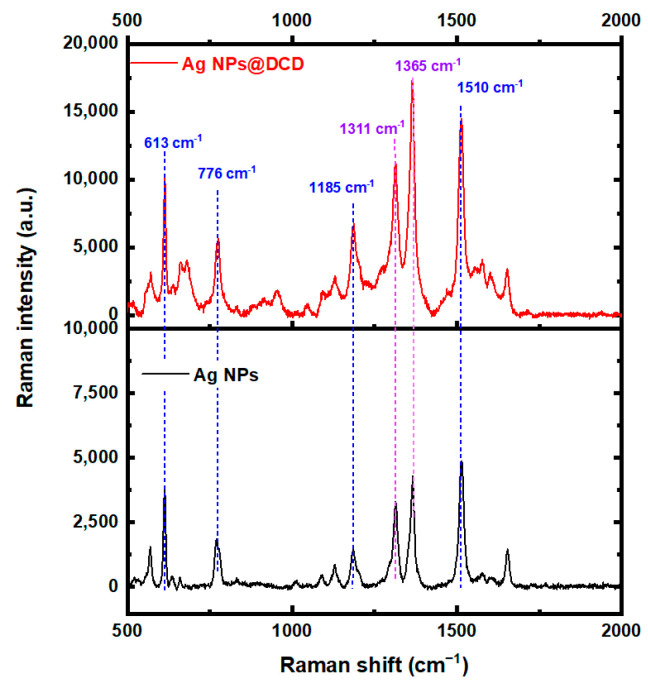
SERS spectra of R6G (1 μM) adsorbed on freshly prepared Ag NPs (curve black) and Ag NPs@DCD (curves red).

Figure 4 depicts the SERS spectra analysis comparing Ag NPs (black curve) to Ag NP@DCD (red curve), utilizing R6G as the Raman reporter. An array of peaks has been identified, specifically strong peaks at 613 cm^−1^ and 776 cm^−1^ that correspond to the C—C—C ring in-plane bending and C-H out-of-plane bending, respectively. Peaks located at around 1311/1365 cm^−1^ are attributed to C—C/C—N stretching, and a strong peak at 1510 cm^−1^ was assigned to the aromatic C—C stretching. It is worth mentioning that Ag NPs@DCD demonstrate enhanced SERS performance compared to the pure Ag NPs, suggesting the formation of a “shell isolated nanoparticle” structure when the surface of Ag NPs is coated with DCD. The thickness of the layer of DCD placed between silver nanoparticles is advantageous for creating more Raman-enhanced “hot spots”. We estimated that the enhancement factor (*EF)* of Ag NPs@DCD SERS signals relative to the ordinary Raman measurement (*EF*_SERS/Raman_; R6G) is about 1.6 × 10^6^.

### 3.3. Enzyme-Mimicking Activities of Ag NPs@DCD

Ag NPs@DCD were assessed for their enzyme-like capabilities in a system with H_2_O_2_ and TMB using UV-Vis spectroscopy. The visible region did not showcase any distinct UV-vis peaks for the TMB mixed H_2_O_2_ solution, as displayed in Figure 3B. Nevertheless, the introduction of Ag NPs@DCD into the catalytic system above demonstrated an obvious absorption at around 652 nm, which was designated to the oxidized TMB. Figure 3B inset provides the relevant digital photographs of the solution. These results verified the peroxidase-mimicking activity of Ag NPs@DCD hybrids in the current study.

**Figure 5:**
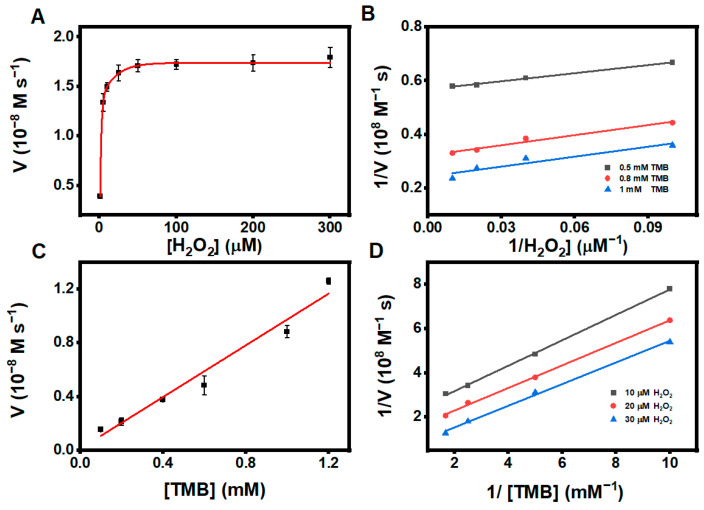
Steady-state kinetic assay of Ag NPs@DCD nanocomposites. (**A**) TMB concentration used was 0.8 mM. (**B**) Lineweaver–Burk plots obtained for H_2_O_2_ with different concentrations (0.5, 0.8 and 1 mM). (**C**) H_2_O_2_ concentration was 20 μM. (**D**) Lineweaver–Burk plots obtained for TMB with serial concentrations of H_2_O_2_ (10, 20 and 30 μM).

To evaluate and understand the catalytic activity of the Ag NPs@DCD, we utilized the steady-state kinetic parameters of the reaction, focusing on the concentration series of H_2_O_2_ and TMB. Figure 5A,C displayed the typical Michaelis–Menten curves for TMB and H_2_O_2_ were obtained by plotting the initial reaction velocity for the substrate concentrations. The Michaelis–Menten equation was applied as follows:

*V*_0_ = *V_max_* [*S*]/*K_m_* + [*S*]

The *V*_0_, *V_max_*, *K_m_*, and [*S*] represent the initial reaction rate and the maximal reaction velocity. These kinetic parameters of Ag NPs@DCD were analyzed with respect to HRP. The *K_m_* value explains the intrinsic properties of a particular enzyme/substrate, where a lower *K_m_* value indicates a stronger affinity of the enzyme for the substrate. The obtained kinetic parameters of Ag NPs@DCD were compared to those of HRP. The *K_m_* value of Ag NPs@DCD for H_2_O_2_ (1.137 mM) was 3.2 times lower than that of HRP (3.700 mM) [44]; this suggests that Ag NPs@DCD possess a higher affinity for H_2_O_2_. Ag NPs@DCD showed 1.4 times lower activity towards TMB (0.307 mM) than HRP (0.434 mM) [44]. This indicates that Ag NPs@DCD have a stronger affinity for H_2_O_2_ than HRP. It is worth noting that Figure 5B,D illustrate a typical ping-pong mechanism with parallel slopes across varying substrate concentrations, resembling that of HRP. The aforementioned results indicate that Ag NPs@DCD showed enhanced catalytic performance as a peroxidase-mimicking agent during the reaction with H_2_O_2_ and TMB.

### 3.4. Conditions for the Experiments

**Figure 6:**
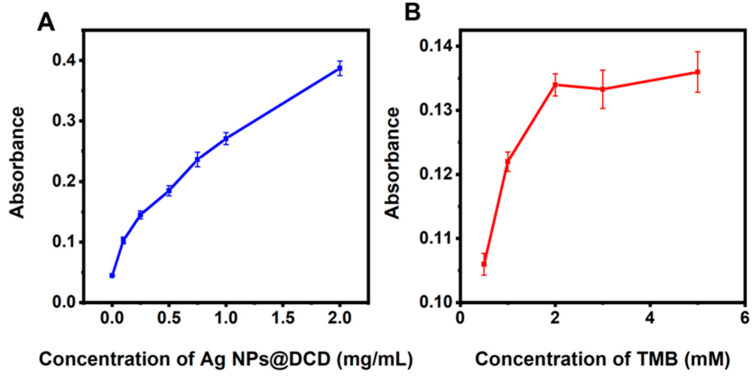
Effect of the concentration of Ag NPs@DCD nanozyme (**A**) and TMB concentration (**B**).

The amount of Ag NPs@DCD utilized, and the concentration of H_2_O_2_ and TMB are vital factors that determine the catalytic performance. The optimization of measuring conditions was conducted by measuring the absorption of oxTMB at 652 nm. According to previous research [35], the catalytic of the enzyme is significantly affected by the temperature during incubation. If the temperature is too low or high, the effectiveness of the enzyme-catalyzed reaction decreases. Hence, we opted for an incubation temperature of 37 °C for this experiment. As depicted in Figure 5A, the absorbance intensity of the reaction solution in the Ag NPs@DCD and TMB reaction increases with the addition of H_2_O_2_. In this reaction system, Ag NPs@DCD can effectively catalyze the decomposition of H_2_O_2_ into the ·OH radicals, leading to the conversion of oxTMB by TMB. However, the absorbance of oxidized TMB ceases to increase at 50 μM. Therefore, we concluded that 50 μM H_2_O_2_ is the optimal reaction concentration for this study. Figure 6A shows an increasing absorbance at 652 nm as the amount of Ag NPs@DCD nanocomposites (ranging from 0 to 2 mg mL^−1^) increase in the presence of TMB (10 mM) and H_2_O_2_ (50 μM). Considering the catalytic efficiency and sensor cost, it is advisable to employ 0.25 mg mL^−1^ of Ag NPs@DCD subsequent experiment. The influence of TMB concentration was also studied within a concentration range of 0.25 to 5 mM. Figure 6B illustrates the present data showing that maximum absorbance is obtained at 2 mM TMB using Ag NPs@DCD (0.25 mg mL^−1^) and H_2_O_2_ (50 μM). Thus, 0.25 mg mL^−1^ and 2 mM TMB were used as the optimized experimental conditions for this work.

### 3.5. Analytical Performance of TMB-Ag NPs@DCD SERS Sensing for Cholesterol Detection

**Figure 7:**
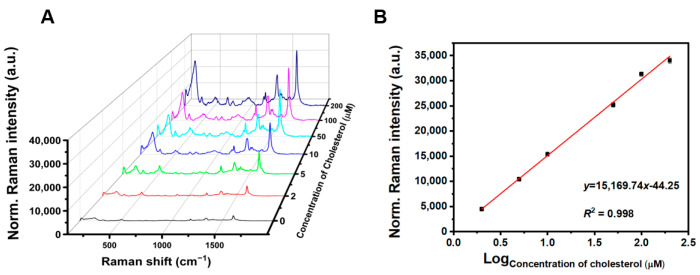
(**A**) SERS spectra of TMB-Ag NPs@DCD solution with various concentrations of cholesterol. (**B**) The SERS intensity with respect to logarithmic concentration of cholesterol at 1605 cm^−1^.

The proposed sensing platform was used to measure various concentrations of cholesterol according to defined experimental parameters. As depicted in Figure 7A, the Raman peak at 1605 cm^−1^ exhibited changes upon the addition of different cholesterol concentrations, establishing a dynamic response range for measuring cholesterol concentration between 2.0 and 200 μM. Additionally, Figure 7B shows the concentration-dependency response, which is explained with the following linear fitting equation: I(_1605 cm_^−1^) = 15,169.74 × Log C_cholesterol_ − 44.25 (R^2^ = 0.998). The calculated detection of limit for this sensing system is 0.8 μM. Table 1 presents a summary of the detection capabilities of various sensing techniques for cholesterol measurement. The results suggest that the method reported in this study showed better performance than some other reported sensing systems in terms of both detection limit and linear range.

### 3.6. Selectivity of the TMB-Ag NPs@DCD SERS Sensing System for Cholesterol Detection

**Figure 8:**
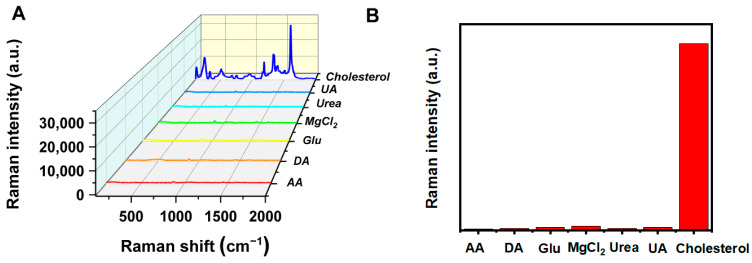
Study on the performance of selectively testing cholesterol with fibrate sensors. (**A**) SERS spectra and (**B**) the characteristic peak of oxTMB at 1605 cm^−1^ indicate to cholesterol (200 μM), Glu (200 μM), AA (200 μM), UA (200 μM), Urea (200 μM), MgCl_2_ (200 μM), and DA (200 μM), respectively.

To test the selectivity of the proposed sensor for cholesterol detection under the defined conditions, we measured the responses of some possible coexisting substances in serum samples, including 200 μM of UA, Uera, MgCl_2_, AA, Glu and DA. Figure 8A,B demonstrate that the Raman intensity of oxTMB remained unaffected by all coexisting substances, except for cholesterol. With the DCD-coated surface of the plasmonic Ag NPs, the stability of the nanoparticle is greatly improved, which can effectively reduce the adsorption of biomolecules on the Ag NPs surface within the complex system and ensure the enzyme-like catalytic efficacy of silver nanoparticles, and at the same time, it shows that Ag NPs@DCD can also play a role as a Raman substrate in Raman signal enhancement. Therefore, the designed SHINERS nanostructure in this work is expected to be widely used in the field of enzyme immunoassay and molecular diagnosis in complex biological samples.

### 3.7. Measurement of Cholesterol in Serum Sample

The TMB-Ag NPs@DCD SERS sensing system was utilized to measure cholesterol in the serum samples. In this study, we employed the standard addition method to detect cholesterol under physiological conditions. The data are presented in Table 2. Using the standard calibration curve depicted in Figure 7B and the customary regression equation, we calculated the cholesterol concentration in the serum samples. This sensing system displays excellent cholesterol measurement sensitivity.

## 4. Conclusions

In summary, the Ag NPs@DCD nanocomposites were prepared using a straightforward ultrasound strategy to form a shell-isolated nanoparticle structure for SERS substrate. The resulting Ag NPs@DCD exhibit oxidase-like behavior and have the ability to facilitate the oxidation of TMB with H_2_O_2_. The as-designed TMB-Ag NPs@DCD SERS sensing system for sensitive cholesterol detection ranging from 2.0 to 50 μΜ, with a low LOD of 0.8 μΜ. The novel biosensor is a label-free assay that does not require a complex labeling procedure. Moreover, TMB-Ag NPs@DCD SERS was shown to be an effective SERS biosensor for detecting cholesterol in rabbit blood samples. At present, the key to clinical diagnosis of this sensor is the stability of the enzyme, and the efficiency of enzyme catalysis directly determines the detection performance of this novel sensor. Nevertheless, the advantages of the present sensor, such as low cost, fast response, high detection sensitivity, and good selectivity, demonstrate its potential application in point-of-care testing (POCT) diagnostics.

## Figures and Tables

**Table 1:** Comparison of detection performances of different sensing systems for cholesterol analysis.

Methods	Sensing System	Linear Range (μM)	LOD (μM)	Refs.
Fluorometry	N-GQDs/CrPic	0–520	0.4	[45]
	b-CD-G-R6G	5–30	5	[46]
	CD-AuNCs	10–100	5.77	[47]
Colorimetry	ChOx/GQDs-TMB-H_2_O_2_	20–600	6	[48]
	ChOx-PB/MWCNT	4–100	3.01	[49]
	PPy NPs	10–100	3.5	[50]
	BNNS@CuS	10–100	2.9	[51]
SERS	AgNPs@MIL-101(Fe)	1–100	0.36	[21]
	HS-β-CD@Au	0.45–22	0.74	[52]
Electrochemial	NPG/SPE	50–6000	8.36	[53]
	ChOx-FG/Gr	50–350	5	[54]
SERS	TMB-Ag NPs@DCD	2–200	0.8	This work

**Table 2:** Total cholesterol monitoring in serum samples with the proposed SERS sensing.

Sample	Added (µM)	Found (µM)	Recovery (%)	RSD (%)
Serum 1		12.87		1.50
	1	13.96	99.80	3.20
	10	21.47	101.90	4.51
	25	39.46	102.80	2.17
Serum 2		10.98		0.98
	1	12.05	99.98	1.46
	10	20.65	102.90	1.58
	25	36.76	101.90	2.68
Serum 3		11.36		1.42
	1	12.50	99.00	1.51
	10	21.98	100.80	2.30
	25	36.07	101.76	1.98

## Data Availability

The raw data supporting the conclusions of this article will be made available by the authors, without undue reservation.
